# mRNA-based therapies: From molecular discovery to clinical revolution

**DOI:** 10.1590/1678-4685-GMB-2026-0026

**Published:** 2026-10-09

**Authors:** Andressa Peres de Oliveira, Fernanda Marques Câmara Sodré, Orlando Louzada-, Mariana Centrone Zylberman, Jamile Ramos da Silva, Luís Carlos de Souza Ferreira, Carlos Frederico Martins Menck

**Affiliations:** 1 Universidade de São Paulo, Instituto de Ciências Biomédicas, Departamento de Microbiologia, São Paulo, SP, Brazil.; 2 Einstein Hospital Israelita, Instituto de Ensino e Pesquisa, São Paulo, SP, Brazil.

**Keywords:** Gene therapy, mRNA-based LNPs, protein replacement, cell transfection

## Abstract

For nearly 50 years, scientists have sought to exploit messenger RNA (mRNA) as a therapeutic tool, overcoming major challenges related to instability, delivery, and inflammatory reactions. A major breakthrough came with the incorporation of modified nucleosides, particularly pseudouridine, during *in vitro* mRNA synthesis, which markedly improved stability, translation efficiency, and tolerability. The clinical success of mRNA technology was demonstrated in 2020 with the development of highly effective and safe COVID-19 vaccines, administered to over a billion individuals worldwide. These achievements inaugurated a new era in molecular medicine. In this review, we revisit the historical development of mRNA delivery into human cells and discuss the emergence of mRNA as a gene-therapy approach. In fact, beyond vaccination, mRNA platforms are rapidly expanding toward protein replacement therapies for genetic disorders and innovative strategies for cancer treatment. We summarize advances in synthetic mRNA design and optimization, delivery vehicles, and administration strategies that enable therapeutic applications. Finally, we highlight current limitations, technological challenges, and future opportunities, emphasizing how mRNA-based technologies have become powerful tools for studying gene function and developing next-generation precision therapies.

## Introduction

Since the discovery of the RNA functions as information messenger, scientists have worked extensively to understand how mRNA could be used to treat human diseases. These efforts faced many challenges until 2008, when Katalin Karikó and Drew Weissman made the fundamental discovery indicating that the incorporation of modified nucleosides into the mRNA molecules (particularly N1-methylpseudouridine and 5-methylcytidine) could overcome exacerbated inflammatory reactions and, together with the development of lipid nanoparticles as a delivery system, rendered these molecules therapeutically viable (Karikó *et al.*, 2005, 2008). In 2020, during the COVID-19 pandemic, the approval of the first mRNA-based vaccines for human use represented a major milestone, highlighting the therapeutic potential of mRNA technology. The vaccination of more than one billion individuals worldwide demonstrated not only high efficacy in protecting humans against specific viral pathogens but also demonstrated the safety and feasibility of using mRNA molecules for broader biomedical applications, including therapies for human genetic and acquired diseases. mRNA-based therapeutics offer several advantages, including the absence of genomic integration, high versatility, and rapid development and production. However, as with any other therapeutic strategy, important challenges remain, particularly regarding cell- and tissue-specific delivery and precise modulation of the immune system (Wei *et al.*, 2023). 

This review focuses on recent advances in the use of mRNA molecules as potential gene therapy tools, highlights current strategies and future perspectives. The application of mRNA molecules as vaccines against cancer is also summarized. In contrast, mRNA vaccines for infectious diseases are addressed primarily from a historical perspective, emphasizing key discoveries related to the delivery of these molecules to cells and organisms. Readers interested in recent developments in vaccines for infectious diseases or cancer are encouraged to consult comprehensive and up-to-date reviews on the topic (Ali, 2025; Quan et al., 2025).

## The history of mRNA delivery systems

The messenger ribonucleic acid (mRNA) was first described in 1961 by François Gros and colleagues (Gros et al., 1961). Seventeen years later, in 1978, mRNA was successfully transfected into cells, when mRNA encoding a globin-like protein was delivered into human epithelial carcinoma (HEp-2) cells using liposomes, resulting in detectable protein translation (Ostro et al., 1978). Subsequently, *in vitro* synthesis of mRNA encoding *Xenopus* β-globin using SP6 bacteriophage RNA polymerase was achieved from complementary DNA (cDNA). Injection of these mRNA molecules into frog oocyte cytoplasm resulted in efficient protein translation. Importantly, this process was shown to depend on the presence of 5’cap modification, whereas deletion of most of the 3’ flanking region, including the poly(A) tail, did not completely abolish protein synthesis (Krieg and Melton, 1984). Further advances demonstrated that *in vitro*-transcribed (IVT) luciferase mRNA could be introduced into NIH 3T3 mouse cells using a lipid-based delivery system, leading to measurable luciferase activity (Malone et al., 1989). Shortly thereafter, direct injection of IVT mRNA (encoding chloramphenicol acetyltransferase, luciferase, and beta-galactosidase) into the skeletal muscle of mice also resulted in successful *in vivo* protein expression (Wolff et al., 1990). Despite these early breakthroughs, progress in mRNA-based therapeutics remained limited for many years due to significant technical challenges, including liability and strong endogenous inflammatory reactions (Sahin et al., 2014).

Building upon these early advances, the pioneer experiments for mRNA therapy were reported in 1992 using Brattleboro rats, a strain that served as a model for diabetes insipidus, due to the inability to produce the hormone vasopressin. Direct intracerebral injection of vasopressin-encoding mRNA restored hormone production and effectively reversed disease symptoms within hours, with therapeutic effects lasting up to five days after treatment (Jirikowski et al., 1992). This study provided one of the earliest proofs of concept that exogenous mRNA could be used to transiently replace defective or absent proteins *in vivo*.

Parallel efforts explored the potential of mRNA as a vaccination platform. In 1993, the IVT influenza virus nucleoprotein mRNA encapsulated into liposomes was shown to induce a robust immune response in mice, stimulating the production of cytotoxic T lymphocytes, essential for eliminating virus-infected cells (Martinon et al., 1993). These findings highlighted the capacity of mRNA to stimulate antigen-specific cellular immunity and suggested its potential application in infectious disease prevention.

Shortly thereafter, the feasibility of mRNA-based cancer vaccines was demonstrated. A capped, polyadenylated, and stabilized mRNA encoding a tumor-associated carcinoembryonic antigen was delivered into mouse fibroblast cells via liposome-mediated transfection, resulting in efficient antigen expression. In pilot *in vivo* experiments, immunization with this mRNA triggered antigen-specific immune responses and protected mice from colon carcinoma cells engineered to express the same antigen (Conry et al., 1995). These results provided early evidence that mRNA vaccination could generate protective antitumor immunity.

Together, these pioneering studies established the conceptual and experimental foundations for mRNA-based therapeutics and vaccination, although widespread application remained limited by technical challenges related to mRNA stability, delivery efficiency, and exacerbated inflammation.

Concurrently, several post-transcriptional nucleoside modifications in RNA were identified, such as the deamination of adenosines and cytidines, which were shown to influence mRNA translation efficiency and regulation (Kiss, 2001). Numerous modified ribonucleotides were subsequently described for the major cellular RNA classes, including tRNAs, rRNAs, mRNA, and snRNAs, most of which are introduced post-transcriptionally (Limbach et al., 1994; Kiss, 2001). These modifications were demonstrated to play essential structural and functional roles. In tRNAs, for example, nucleotide modifications promote accurate anticodon-codon pairing and enhance both the efficiency and fidelity of translation (Agris, 1996; Kiss, 2001).

In the early 2000s, fundamental discoveries in understanding mRNA inflammatory reactions emerged from studies of Karikó and Weissman. Their work demonstrated that IVT RNA molecules activate human Toll-like receptor (TLR) 3, thereby triggering innate immune responses (Karikó *et al.*, 2004). Similarly, bacterial RNA was also shown to stimulate innate immunity and inflammation, while mRNA and tRNA derived from eukaryotic sources failed to activate cultured human dendritic cells (DC) (Koski et al., 2004). TLRs are key initiators of adaptive immune responses, promoting T-cell differentiation into T helper 1 effector cells against microbial infections through recognition of foreign nucleic acids (Medzhitov, 2001). Importantly, activation of these receptors by exogenous mRNA significantly limits protein expression due to cell death following cellular transfection, representing a major obstacle for therapeutic applications. Subsequent studies by Karikó and Weissman led to a seminal discovery demonstrating that the incorporation of modified nucleosides-including 5-methylcytidine (m5C), N6-methyladenosine (m6A), 5-methyluridine (m5U), 2-thiouridine (s2U), and pseudouridine-during *in vitro* mRNA synthesis markedly suppresses TLR-mediated inflammatory reactions (Karikó *et al.*, 2005). Their work furthermore showed that mitochondrial RNA (mtRNA) stimulates monocyte-derived dendritic cells (MDDCs), whereas mammalian tRNA did not induce detectable levels of tumor necrosis factor alpha (TNF-α). This difference is likely explained by the structural similarity between mitochondrial and bacterial RNA, both of which contain relatively few nucleoside modifications and therefore act as potent DC activators. In contrast, tRNAs are highly modified and show little or no activity of DC activation (Karikó *et al.*, 2005). Further advances demonstrated that mRNA containing pseudouridine exhibits significantly enhanced translational capacity compared to unmodified transcripts, when introduced in mammalian cells, cell-free lysates, or following intravenous administration into mice. Importantly, only unmodified mRNA induced strong immunogenic responses, including high levels of interferon alpha production (Karikó *et al.*, 2008). In Figure 1, a historical timeline for these discoveries, which turned possible the use of mRNA for vaccine and therapy, is presented.

Collectively, these findings established that incorporation of modified nucleosides, particularly pseudouridine, during *in vitro* mRNA synthesis was critical for reducing innate immune recognition, cell death and activation of deleterious inflammatory responses, which resulted in enhanced expression of proteins encoded by the exogenous mRNA and, thereby, enabling the development of effective therapeutic mRNA platforms. Figure 2 illustrates the biological outcomes of transfection with RNA molecules synthesized with pseudouridine, namely elimination of innate inflammatory responses, and improvement of translation efficiency.

**Figure 1 -:**
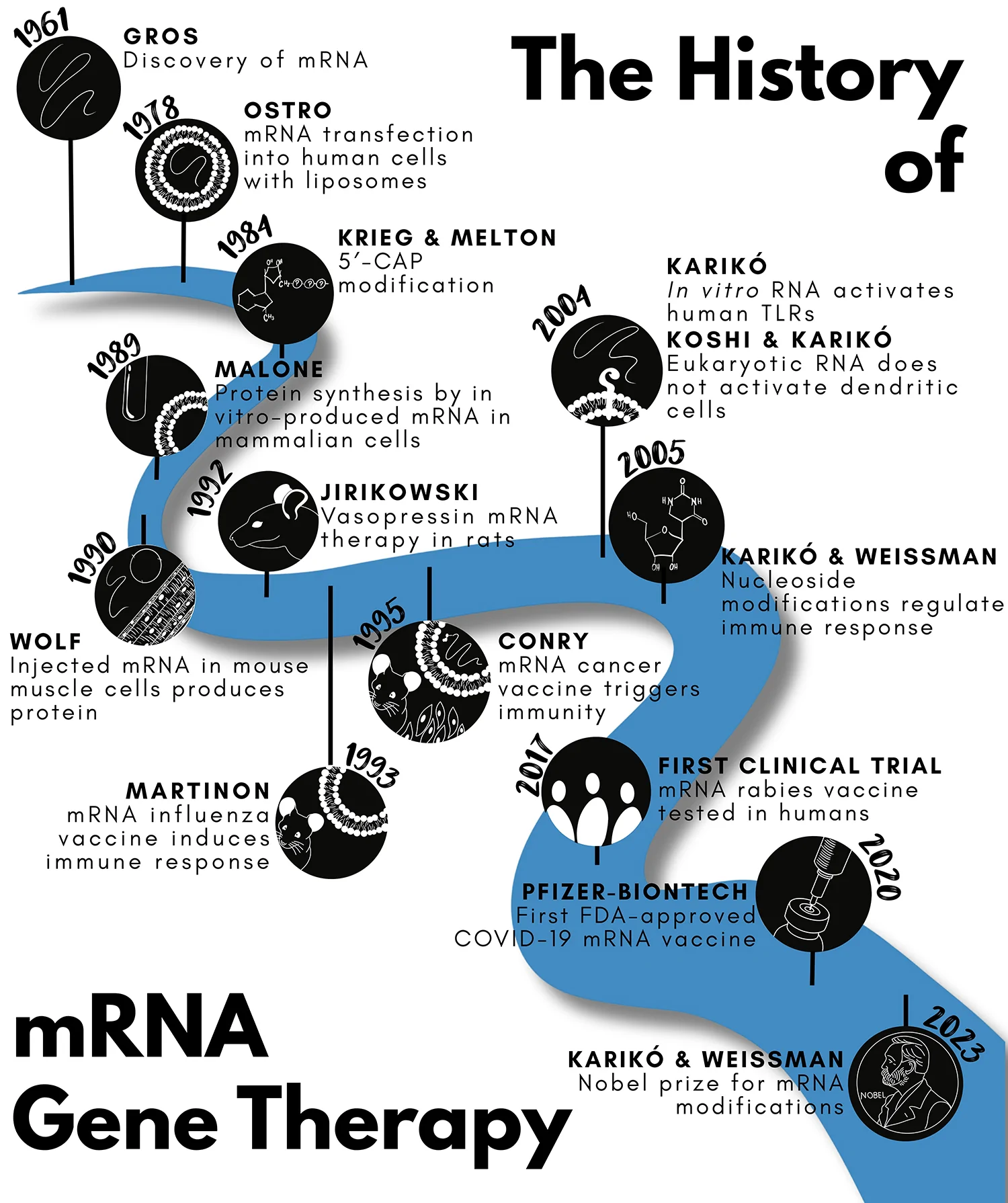
The timeline history of mRNA gene therapy.

**Figure 2 -:**
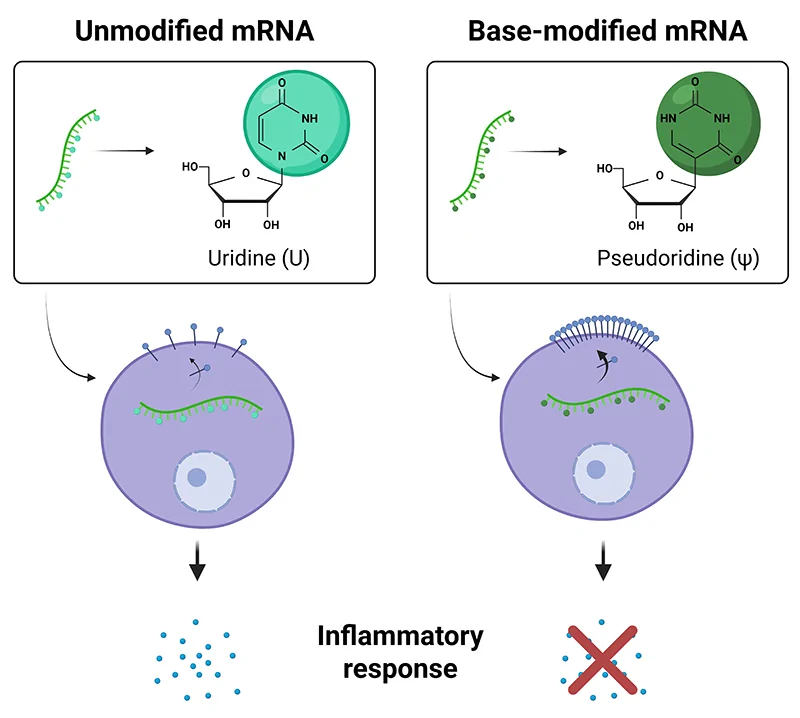
Representative scheme of the biological outcome of uridine substitution with pseudouridine. Replacement of uridine with pseudouridine during *in vitro* mRNA synthesis provides significant advantages when the mRNA is introduced into cells. These benefits include higher translational efficiency, leading to increased protein production, as well as a reduced inflammatory response. Figure adapted from the Nobel Prize announcement website. https://www.nobelprize.org/prizes/medicine/2023/advanced-information/.

As a consequence of the landmark discoveries made by Katalin Karikó and Drew Weissman, along with their broader outstanding contributions to the development of nucleoside-modified mRNA technology, they were awarded the 2023 Nobel Prize in Physiology or Medicine (https://www.nobelprize.org/prizes/medicine/2023/). Their work was fundamental by enabling the development of effective mRNA vaccines**,** including those used against COVID-19. 

The first-in-human clinical trial for an mRNA vaccine encoding the rabies virus glycoprotein (CV7201) was conducted before any signs of the COVID-19 pandemic and published in 2017 (Alberer et al., 2017). In this study, female and male volunteers with no prior history of rabies vaccination received escalating doses administered intradermally or intramuscularly using needle-syringe or needle-free devices, followed by a booster dose after one year. This trial represented the first demonstration of an effective mRNA-based vaccine tested in humans. The vaccine successfully induced antibody responses against the viral antigen and proved to be safe and well-tolerated (Alberer *et al.*, 2017). 

Of note, in 2018, the U.S. Food and Drug Administration (FDA) approved the first lipid nanoparticle (LNP)-based therapy carrying a small interfering RNA (siRNA) (patisiran, Onpattro^®^) (Akinc et al., 2019). This drug, indicated for the treatment of polyneuropathy associated with hereditary transthyretin-mediated amyloidosis in adults, inhibits the hepatic synthesis of transthyretin (mutant and wild-type). It acts by specifically binding to a genetically conserved sequence in the 3’ untranslated region of transthyretin messenger RNA (Hoy, 2018). This approval marked the first regulatory validation of an LNP system designed to deliver an RNA molecule in humans and demonstrated that such platforms could safely and effectively enable systemic nucleic acid delivery. Indeed, incorporation of the mRNA molecules into ionizable LNPs conferred both increased stability to the molecules and, simultaneously, offer a highly efficient approach to introduce mRNA molecules into the cytoplasm of transfected cells. These advances were critical for clinical translation and later supported the development and approval of mRNA-based vaccines for human use. 

In December 2020, the biotech companies Pfizer-BioNTech and Moderna received emergency use authorization for the COVID-19 mRNA-based vaccines from the U.S. FDA and from the European Medicines Agency (EMA). In both vaccines, the nucleoside-modified mRNA encoding the SARS-CoV-2 spike protein is administered intramuscularly using lipid nanoparticle-formulation. These groundbreaking vaccines played a pivotal role in mitigating the impact of the COVID-19 pandemic. Their rapid development, high efficacy, and adaptability to emerging viral variants established a new paradigm for vaccine design and highlighted the potential of mRNA platforms for combating future infectious diseases and other medical conditions (Lamb, 2021). 

## mRNA-based therapy for protein replacement 

Several human genetic disorders result from mutations leading to deficiencies in the activity or the production of specific enzymes. A transformative therapeutic strategy for many of these conditions has been enzyme replacement therapy (ERT), which involves the application of recombinant forms of the missing enzyme to patients, with important clinical benefits. However, ERT presents several limitations, including reactogenicity, limited penetration into the central nervous system, and variable transfection efficacy for skeletal muscle. In addition, the high costs of the treatment substantially restricted its widespread clinical use (Choi et al., 2025).

Following the demonstrated success and safety of mRNA-based vaccines, the therapeutic use of mRNA that efficiently expresses the deficient enzyme has emerged as a promising alternative for the treatment of certain genetic disorders. Thus, the mRNA-based protein replacement represents an innovative strategy in which synthetic IVT mRNA is delivered to the patient cells to direct the synthesis of functional therapeutic proteins employing the host cellular translational machinery (Żak and Zangi, 2025). This approach offers potential advantages, including transient protein expression, avoidance of genomic integration, and flexible manufacturing. In this section, key studies of mRNA-based therapeutics for genetic disorders are presented.

Considering hematological diseases, hemophilia A and hemophilia B are caused by deficiencies in factors VIII and IX, respectively (Sidonio and Malec, 2021). Preclinical studies evaluating mRNA-based therapies have been conducted to explore treatments for those disorders. For hemophilia B, an mRNA-drug encapsulated in LUNAR lipid nanoparticles (LNPs; LUNAR- a proprietary mRNA delivery platform developed by Arcturus Therapeutics, San Diego, CA, USA) encoding human factor IX (hFIX) was administered in immunocompetent hemophilic mice. High circulation levels of hFIX protein were detected after repeated administrations, demonstrating sustained protein expression (Ramaswamy et al., 2017). Similarly, studies investigating mRNA-based therapy for hemophilia A demonstrated that intravenous injection of LNP encapsulated luciferase mRNA, resulted in detectable hepatic luminescence within four hours, with no toxicity even after multiple administrations. Based on these findings, the mRNA encoding factor VIII protein was subsequently evaluated (Chen et al., 2020). This approach successfully restored biological activity, and production of therapeutic factor VIII (FVIII) protein levels was detected, above therapeutic levels, over five days. Repeated injections of FVIII-mRNA prolonged the duration of FVIII expression (Chen *et al.*, 2020). 

The Ornithine Transcarbamylase (OTC) deficiency (OTCD) is an X-linked genetic disorder, characterized by severe disease onset during the neonatal period, most frequently in males, although the post-neonatal manifestations may occur in both sexes (Lichter-Konecki *et al.*, 2022). OTC is a key enzyme in the urea cycle, a metabolic pathway responsible for ammonia detoxification. Deficiency of OTC leads to an ammonia build up in the blood (hyperammonemia), which is neurotoxic in affected individuals. To address these disorders, LNP formulations encapsulating OTC mRNA have been developed as a hepatocyte-targeted delivery system for OTC intracellular enzyme replacement therapy. This delivery system promotes cellular uptake and prevents endosomal escape, protecting mRNA against nucleases, enabling efficient expression in liver cells. High expression of OTC protein was detected in murine liver, resulting in a prolonged benefit for the murine OTCD model (Prieve et al., 2018), including significant reduction of hyperammonemia in the blood (Yamazaki et al., 2023).

Importantly, phase 1/2 clinical trials for OTCD were conducted with mRNA-based therapy with a proprietary LNP (ARCT-810, from ARCTURUS Therapeutics). The results indicated that multiple doses were safe and well tolerated. The treatment also produced encouraging clinical outcomes, including a significant reduction of glutamine levels (a biomarker of this condition), bringing them to normal range levels (Richard et al., 2024). 

Type I glycogen storage diseases (GSD-I) are rare inherited metabolic disorders comprising two autosomal recessive conditions: GSD-Ia, resulting from a deficient activity of glucose-6-phosphatase-α (G6Pase-α or G6PC), and GSD-Ib, caused by impaired function of the glucose-6-phosphate transporter (G6PT or SLC37A4) (Chou and Mansfield, 2023). These enzymes are key for glucose homeostasis, catalyzing the translocation of glucose-6-phosphate (G6P) from the cytoplasm into the endoplasmic reticulum and its subsequent hydrolysis to glucose, which is then released into the bloodstream (Chou and Mansfield, 2023). Patients exhibit life-threatening hypoglycemia and are at increased risks of hepatocellular adenoma (HCAs)/carcinoma (HCCs) and progressive renal disease (Chou and Mansfield, 2023). 

Preclinical studies have explored mRNA-based therapeutic approaches for GSD-I. LNP-encapsulated *G6PC* mRNA variants were designed and delivered to the liver in a murine model, with the expression of an active variant of G6Pase-α enzyme. This mRNA-based drug improved fasting blood glucose levels and caused reduction in liver mass over a 12 days treatment period (Roseman et al., 2018). Subsequent experiments demonstrated that LNPs carrying mRNA encoding human G6Pase-α were capable of correcting hypoglycemia and reducing the risk of hepatic tumor development in a murine model (Cao et al., 2021). Building in these promising preclinical results, Moderna launched a phase 1/2 clinical trial in 2022 to assess the safety, tolerability, pharmacokinetics, and pharmacodynamics of mRNA-3745- an LNP encapsulated, codon-optimized G6PC mRNA (coG6PC-S298C)-in patients with GSD-Ia. The study involves a single intravenous dose administration, designed to primarily target mRNA delivery to the liver. Initial results indicate that the drug is safe and showed promising signs of clinical benefits (Chou and Mansfield, 2023).

Another metabolic genetic disorder that has an ongoing development of a mRNA-based therapy is phenylketonuria (PKU) (van Spronsen et al., 2021). This is an inherited, autosomal recessive metabolic disorder of phenylalanine metabolism that leads to neurological complications if untreated, affecting approximately 1 in 10,000 newborns globally. The disease results from deficiency or dysfunction of phenylalanine hydroxylase, leading to toxic accumulation of phenylalanine and its metabolites. The mRNA 3210 was developed to restore functional enzyme expression. Preclinical studies demonstrated favorable pharmacokinetic and pharmacodynamic (PK/PD) effects, eliciting measurable functional protein activity production and reducing relevant biomarker levels compared to control-treated animals (Baek et al., 2024). Based on these encouraging findings, Moderna initiated a phase1/2 test of the mRNA 3210 in individuals with phenylketonuria to evaluate its safety, tolerability, pharmacokinetics, and pharmacodynamics. However, the trial was withdrawn before the enrollment of any patient, most likely because of a strategic business decision, prioritizing other programs.

Another rare inherited metabolic disorder under investigation for mRNA-based for protein replacement therapy is methylmalonic acidemia (MMA). This disorder is caused by defects in the activity of methylmalonyl-CoA mutase (MUT) or its cofactor adenosylcobalamin (a biological form of vitamin B12), which is associated with high morbidity and mortality in affected children (Almási et al., 2019). A pseudouridine-modified codon-optimized mRNA encoding the human MUT encapsulated within a LNP was administrated intravenously in murine models, resulting in high hepatic expression and enzymatic activity of MUT, with no evidence of toxicity or inflammation (An *et al.,* 2017). Later, the same research group assessed long-term efficacy and safety through repeated intravenous administrations over two 12-week dosing cycles. The MUT protein was consistently detected in the liver, in a dose-dependent manner, with good tolerability and no significant adverse effects observed (An *et al.*, 2019). The first clinical evaluation of this therapeutic strategy was conducted in a phase 1/2 clinical trial sponsored by Moderna. In 2021, Moderna launched a phase 1/2 study evaluating mRNA-3705, an optimized version of the original MUT-encoding mRNA (Coughlan et al., 2024). The treatment was well tolerated, producing promising outcomes, including reductions in disease-associated biomarkers and clinical events. Participants who completed these initial studies were subsequently enrolled in an ongoing extension study (NCT05295433) designed to assess the long-term safety and efficacy of mRNA-3705 therapy.

Propionic acidemia (PA) is an inherited metabolic disease caused by mutations in the *PCCA* or *PCCB* genes, which encode the α and β subunits of propionyl-CoA carboxylase (PCC), respectively. Deficiency of PCC activity disrupts the propionyl-CoA metabolism and is associated with severe metabolic instability and neurological dysfunctions (Chen et al., 2025). An innovative approach with the two mRNAs encoding for both human subunits (hPCCA and hPCCB) encapsulated in an LNP was evaluated in a hypomorphic murine model of PA (Jiang et al., 2020). Mice treated with the dual mRNA formulation exhibited higher PCC activity in the liver when compared with animals receiving either mRNAs alone. This treatment also resulted in a sustainable reduction of toxic metabolites associated with the disease, following long term repeated dosing at three and six-months (Jiang *et al.*, 2020). A phase 1/2 clinical trial, sponsored by Moderna, is currently evaluating a similar dual mRNA therapeutic candidate, mRNA-3927. The mRNA-drug is administered intravenously, and has demonstrated good tolerability, with no significant toxicity reported, with clinical benefits, such as a 70% reduction in the risk of metabolic decompensating events (Koeberl et al., 2024). Collectively, these results support the feasibility of chronic administration of multiple mRNA molecules for protein replacement in genetic disorders.

Niemann-Pick type C (NPC) is an ultrarare lysosomal storage disorder (LSD) characterized by the accumulation of endo-lysosomal cholesterol and glycosphingolipids due to the mutations in the *NPC1* and *NPC2* genes, leading to loss of protein functions, affecting several organs, including the brain, liver, and spleen (Las Heras et al., 2023). An mRNA encoding the NPC1 protein has been shown to restore protein expression and rescue the pathogenic phenotype in cultured fibroblasts derived from an NPC1 patient. Moreover, optimization of mRNA secondary structure, through codon optimization and base modification, significantly enhanced protein NPC1 expression (Furtado et al., 2022). 

Most of the advances discussed above have focused on mRNA delivery to the liver, whereas targeting other tissues remains a significant challenge. Several studies have investigated mRNA delivery to the lungs to treat genetic pulmonary disorders such as surfactant protein B (SP-B) deficiency, asthma, and cystic fibrosis (CF) (Sahu et al., 2019; Gill et al., 2025). Progress in different delivery technologies has enabled the development of systems that enhance mRNA stability and translation efficiency in pulmonary tissues. In particular, inhalation-based delivery methods such as nebulizers and inhalers provide direct access to the respiratory tract, allowing bypass of hepatic uptake while exploiting the lung’s large surface area and thin epithelial barrier. Nevertheless, biological barriers, including mucus, surfactants, and local immune responses, still represent important obstacles to efficient mRNA delivery (Biesel et al., 2025). Notably, spray-dried LNP formulations have emerged as promising platforms, improving stability and facilitating mRNA-drug delivery to both upper respiratory tract and lung tissues (Friis et al., 2023).

In summary, mRNA-based protein replacement therapy holds substantial potential for the treatment of rare genetic disorders, immune-mediated conditions through the expression of antibodies and cytokines, and a broad range of protein deficiency diseases. Unlike other therapeutic modalities, protein replacement strategies require repeated and chronic administration, sustained expression of therapeutic proteins, and efficient cell- and tissue-specific delivery. Continued advances in delivery systems, mRNA stabilization, and translational efficiency will be critical for expanding the clinical applicability of this therapeutic approach (Wei et al., 2023). 

## Comparison of RNA therapy with traditional protein replacement therapy

Both mRNA and protein replacement therapies aim to restore functional proteins (Forenzo et al., 2025). However, they achieve this through fundamentally different biological and manufacturing pathways. Instead of introducing external proteins directly into cells as traditional protein replacement, mRNA therapy provides cells with genetic blueprints. This allows the cell’s own internal machinery to manufacture the necessary proteins for therapy (Vavilis et al., 2023; Forenzo *et al.*, 2025). The detailed comparison between RNA Therapy and traditional Protein Replacement Therapy is presented in Table 1. 

**Table 1 -:** Comparison of mRNA-based and protein replacement therapy.

	mRNA Therapy	Protein Replacement Therapy
Mechanism of Action	Delivery into the cytosol; host ribosomes synthesize the functional protein	Provides ready-to-use recombinant proteins for immediate therapeutic action at the target site
Duration of effect	Requires hours to be translated (peaks at 24-48 hours) and degrades via normal cellular processes within days to weeks	Short-to-medium duration: Acts instantly but requires frequent, repeated dosing due to rapid clearance from systemic circulation
Dosage	A single mRNA can produce thousands of protein copies, making precise dosing difficult to control across different patients	Pharmacokinetics and active doses can be titrated precisely during administration
Manufacturing	Synthesized via *in vitro* transcription (IVT). The manufacturing platform remains identical; only the sequence changes, enabling short development cycles and lower costs	Requires custom cultures (bacterial or mammalian expression systems), complex folding, and extensive, highly expensive purification protocols for every distinct protein
Immunogenicity and Safety	Modified mRNA can not trigger innate immune pathways; Bypasses genomic integration risks	Exogenous proteins can trigger a adaptive immune response
Storage	Often requires ultra-low temperature storage	Standard refrigeration (2 °C to 8 °C)
Administration - Delivery Requirements	mRNA is highly unstable and instantly degraded by extracellular RNases. It must be encapsulated in a delivery vehicle, most commonly Lipid Nanoparticles (LNPs) or polymeric carriers, to survive circulation and enter cells	The mature recombinant protein is generally stable enough to be injected directly into the bloodstream or tissue without a protective synthetic carrier

## Pharmacokinetics of mRNA *in vivo*

IVT mRNA, delivered *in vivo*, yields a transient therapeutic protein expression. In animals and humans, expression typically begins within hours, peaks within 1 to 3 days, and gradually decreases within 1 to 2 weeks. However, this depends on the animal model, the site of injection, the use of modified nucleosides, and whether the protein is intended to be secreted or retained inside the cell (Vavilis et al., 2023). In a study of mRNA-LNP expression kinetics across various injection routes in mice, protein expression (luciferase) peaked rapidly around 24 hours post-injection up to 10 days (Pardi et al., 2015). When evaluating therapeutic monoclonal antibodies (mAbs) delivered via mRNA-LNPs in non-human primates, functional antibodies were robustly expressed in serum within 24 hours, though the systemic clearance of the protein itself can extend its presence in the blood for up to 11 to 15 days (Tkaczyk et al., 2024). In humans, clinical observations show that protein can be detected in specific tissues (like the lymph nodes) or circulation for up to 30 days, and occasionally longer depending on the protein’s native half-life (Boros et al., 2024).

## mRNA-based cancer therapy 

The fight against cancer has gained an important ally with the advent of mRNA-based vaccines. Throughout life, the immune system is capable of eliminating malignant cell precursors, preventing cancer progression, until carcinogenic cells obtain genetic or epigenetic modifications enabling them to escape the immune surveillance, leading to the emergence of immune evasive phenotypes (Galassi et al., 2024). Cancer cells can escape immune control through three primary mechanisms: avoidance of immune detection, inhibition of immune effector cells (either directly or indirectly), and cytoprotection mechanisms, which defend cancer cells to the cytotoxic active immune cells (Kim and Cho, 2022; Galassi *et al.*, 2024). Overcoming these limitations requires strategies capable of enhancing immune recognition and destruction of tumor cells, and mRNA cancer vaccines have emerged as a promising therapeutic alternative. Basically, the mRNA-based vaccination aims to induce or boost the immune responses capable to recognize and destroy neoplastic cells (Vishweshwaraiah and Dokholyan, 2022).

The accumulation of somatic DNA mutations is a fundamental driver to cancer (Sahin and Türeci, 2018). These mutations can generate tumor-specific neoepitopes (neoantigens) that are recognized by T cells as foreign, making them attractive targets for cancer vaccines. Since most tumors harbor unique set of mutations, with relatively few mutations shared between patients, technological advances in genomics, bioinformatics, and cancer immunotherapy now enable rapid identification of mutations through sequencing, allowing the development of personalized therapeutic for vaccines (Sahin and Türeci, 2018). This is the case of BNT122 [autogene cevumeran (RO7198457)], developed through a collaboration between BioNTech and Genentech. This personalized mRNA-based LNP vaccine is designed to stimulate immune responses against a patient’s unique tumor neoantigens. Clinical trials phase 1/2 are currently underway to evaluate the therapeutic potential of autogene cevumeran, either as a monotherapy or in combination with immune checkpoint inhibitors accross multiple cancer types, including melanoma, colorectal cancer, non-small cell lung cancer, bladder cancer, colorectal cancer, triple negative breast cancer, renal cancer, head and neck cancer, and other solid cancers (BioNTech, 2023).

Similarly, the mRNA-4157 (V940), a personalized neoantigen mRNA-based therapeutic vaccine (developed by Moderna in collaboration with Merck & Co), is currently being evaluated in combination with pembrolizumab in patients with high-risk melanoma. Phase 2b clinical trial results indicate that combining mRNA-4157 with pembrolizumab improved recurrence-free survival compared to pembrolizumab monotherapy, while maintaining a tolerable safety profile (Weber et al., 2024). The mRNA-4157 has initiated a phase 3 clinical trial. 

Additional innovative strategies have been explored to enhance the therapeutic efficacy of mRNA-based cancer vaccines. For instance, polymeric nanoparticle platforms delivering mRNA encoding anti-PD-1 antibodies, inhibiting the immune checkpoint signaling, to potentiate the efficiency of neoantigen-based mRNA vaccine for pancreatic ductal adenocarcinoma. This approach increased therapeutic efficiency by activation and infiltration of tumor-specific T lymphocytes (Zhu et al., 2025).

Another innovative strategy involves an mRNA-based LNP encoding the KRAS G12D neoantigenic epitope combined with cyclic GMP-AMP (cGAMP), a stimulator of interferon gene signaling (Xu et al., 2025). KRAS represents one of the most frequently mutated oncogenes in human cancers, with the G12D mutation as the most prevalent across several carcinomas and adenocarcinomas (Zeissig et al., 2023). In preclinical models, intravenous administration of the cGAMP/mRNA-KRAS/LNP formulation suppressed metastatic tumor growth, prolonged animal survival, and induced a robust immune response primarily mediated by CD8+ cytotoxic T cells (Zeissig *et al.*, 2023). 

Another case of success was described for glioblastoma in a Phase 1 clinical trial. Intravenous administration of personalized mRNA lamellar particle aggregates (RNA-LPA) encoding tumor-specific glioblastoma antigens was evaluated in patients with newly diagnosed, unmethylated glioblastoma. Following the fourth vaccine dose, patients showed an increase in immunological activation, and one month later, the biopsy revealed no detectable viable tumor in the treated regions (Ghiaseddin et al., 2023).

Human papillomavirus (HPV) infection is responsible for most cervical, anal, and oropharyngeal cancers, with cervical cancer remaining a leading cause of cancer-related mortality among women globally (Jensen et al., 2024). The viral oncoproteins E6 and E7 play a pivotal role in the development and progression of HPV-associated cancers and premalignant lesions, making them targets for therapeutic vaccine development (Movahed et al., 2024). Nevertheless, development of effective therapeutic vaccines for the treatment of lower genital tract HPV infections remains an unmet challenge (Movahed *et al.,* 2024). The therapeutical potential of mRNA-based vaccines for HPV encoding E6 and E7 proteins were tested in a murine model (C57BL/6J). Intramuscular and subcutaneous administration of these vaccines triggered robust T cell-mediated immune responses and suppressed tumor progression (Lee et al., 2023). 

Similarly, mRNA vaccines encoding the HPV-16 E7 oncoprotein genetically fused with the glycoprotein D of type 1 Herpes Simplex virus (HSV-1) demonstrated strong immunogenicity and therapeutic efficacies at preclinical conditions (da Silva et al., 2023). For the first time, the study compared side-by-side the anti-tumor effects of three mRNA vaccines encoding the same target antigen but produced with three different technological platforms: non-replicative mRNA with non-modified nucleosides, non-replicative mRNA with modified nucleosides, and auto-replicative mRNA capable to multiplicate within the transfected cells (da Silva *et al.*, 2023). The results demonstrated that the three mRNA vaccines activated similar levels of antigen-specific T CD8^+^ cytotoxic responses leading to complete remission of tumors in treated animals even after a single administration of the vaccines. At the tested condition, the mRNA anti-cancer vaccines proved to be superior to similar vaccines against tumors induced by HPV but based on naked DNA or purified protein admixed with adjuvants (da Silva *et al.*, 2023). Notably, mice immunized with the vaccines mounted a long-lived immunological memory capable to prevent tumor relapses after the initial treatment opening perspectives for a new generation of anti-tumor therapies (da Silva *et al.*, 2023). 

Another promising immunotherapeutic strategy involves chimeric antigen receptor (CAR) T-cells, which are genetically modified immune T lymphocytes designed to recognize and kill cancer cells through antigen-binding receptors (Wu et al., 2025). Conventional CAR T-cell therapy requires an *ex vivo* manufacturing process involving T-cell isolation, activation, modification, expansion, and reinfusion, typically demanding several weeks to complete (Wu *et al.*, 2024). This individualized and labor-intensive workflow is not only time-consuming but also costly, and broad patient access remains limited. Viral vectors and CRISPR-based gene-editing are often used to transfer CAR transgenes into immune cells. However, these approaches carry risks related to genome integration and long-term genetic alterations. Because mRNA-based transfection excludes the risk of genome integration, *in vitro* transcribed mRNA encoding CAR constructs can be transiently delivered into T-cells via electroporation or nanoparticle-based carriers, and represent a landmark linking CAR therapy and mRNA technology. These constructs can be transfected *ex vivo* or directly administered *in vivo*. Although mRNA-mediated expression is transient, this approach enables multiple dosing, reduces manufacturing time and costs, with lower toxicity (Qin et al., 2025). 

In summary, the central principle of mRNA-based cancer therapy is to train the immune system against the tumor-speciﬁc antigens, killing the cancer cells with high specificity. The range of tumors currently under investigation using mRNA-based LNP platforms, either alone or in combination with checkpoint inhibitors, is extensive. Clinical trials, including several in advanced phases, have shown promising results in both safety and efficacy. Readers interested in specific tumors models, preclinical developments, and clinical trials involving mRNA-based therapeutic vaccines are encouraged to consult specialized reviews on the subject (Wei et al., 2023).

## Structural and functional optimization of synthetic mRNAs for therapeutic applications

For therapeutic applications, including vaccines and therapeutic mRNA-delivered gene editing systems, such as CRISPR-Cas, synthetic mRNAs require structural optimization to maximize translational efficiency, enhance stability, and minimize unwanted immunogenicity. These improvements focus on five key features: the 5′ cap, nucleotide modifications, poly(A) tail design, untranslated regions (UTRs), and codon usage. In Figure 3, a scheme for the step-by-step *in vitro* mRNA synthesis is presented.

**Figure 3 -:**
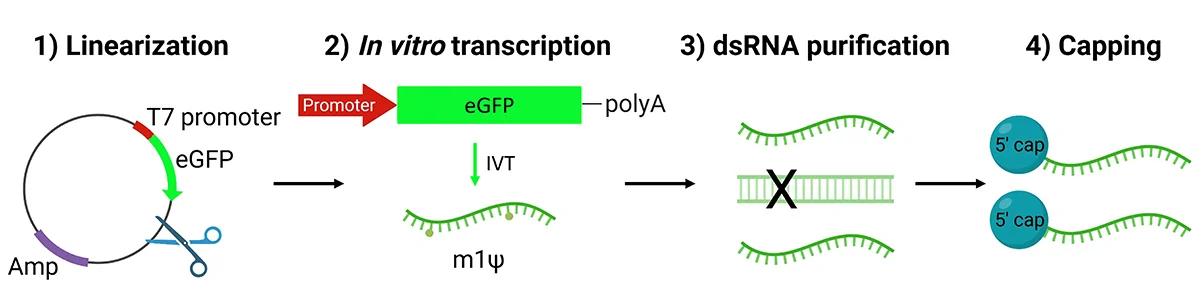
Representative scheme of *in vitro* mRNA synthesis. The process includes (1) plasmid linearization, (2) *in vitro* transcription by the T7 phage RNA polymerase, with incorporation of modified nucleotides such as pseudouridine (m1Ψ), (3) purification to remove double-stranded RNA contaminants, and (4) capping reaction to introduce a 5′ cap structure. These steps result in functional mRNA suitable for experimental or therapeutic applications.

Therapeutic mRNAs commonly employ a Cap1 structure, which enhances translation and reduces innate immune activation by closely mimicking endogenous transcripts (Devarkar et al., 2016). In parallel, replacement of uridine with N1-methyl-pseudouridine (m¹Ψ) has become a standard feature, improving ribosome processivity while minimizing immune sensing and enhancing structural instability (Figure 2) (Karikó et al., 2005). The impact of these chemical modifications was underscored by the superior efficacy of Pfizer-BioNTech and Moderna vaccines compared to the unmodified CureVac formulations (Morais et al., 2021). 

The 3′ poly(A) tail is equally critical, contributing with mRNA stability, translation initiation, and protection from exonucleolytic degradation. Poly(A) tails of approximately 100-120 nucleotides are considered optimal, enhancing stability and translation via interactions with poly(A)-binding proteins and the cap-binding complex, promoting formation of a closed-loop mRNP structure (Holtkamp et al., 2006).

UTRs further modulate mRNA behavior post-transcriptionally. The 5′-UTR influences ribosomal scanning and start codon recognition, and inclusion of elements like the α-globin UTR or optimized Kozak motifs significantly boost translation (Trepotec et al., 2019). Incorporation of 5′ UTR from Tobacco etch virus (TEV) enhances translation by supporting cap-independent initiation through structured AU-rich elements that promote ribosome recruitment (Gallie et al., 1995). This enables efficient protein synthesis even without canonical cap recognition. The addition of a 5′ cap further increases stability and translation efficiency, resulting in higher protein production *in vivo* (Karikó et al., 2008). In a combinatorial screen of synthetic UTRs, translation efficiency, rather than RNA stability, was demonstrated to be the primary determinant of protein output, with the 5′-UTR playing a dominant role (Asrani et al., 2018). The 3′-UTR, in contrast, regulates stability through interactions with RNA-binding proteins and microRNAs. Reducing miRNA-binding site density or incorporating stabilizing elements can prolong transcript half-life and enhance protein expression (Mayr and Bartel, 2009). Lastly, codon optimization improves translation by aligning mRNA coding sequences with the host’s codon usage bias. This strategy enhances ribosome flow, minimizes stalling, and limits secondary structures that could impair translation or trigger degradation (Paremskaia et al., 2024).

## mRNA therapy improvements, delivery systems, and administration routes

Despite the remarkable therapeutic promise of mRNA-based formulations, several obstacles still limit their broader clinical application, including intrinsic instability, potential immunogenicity, and suboptimal delivery strategies. Enzymatic degradation during production can be minimized by nuclease-free working conditions, while structural modifications, as mentioned above, enhance mRNA stability, translational efficiency, and reduce innate immune activation.

For effective *in vivo* function, mRNA therapeutics require delivery systems that are safe, effective, stable, and, ideally, scalable for large-scale production. An optimal carrier must protect mRNA from external degradation, evade immune surveillance, reach target cells, promote cellular uptake, enable endosomal escape, and minimize toxicity. Importantly, delivery systems must be tailored to the therapeutic goal. For example, immune activation is desirable in mRNA vaccines but undesirable in protein-replacement therapies that require repeated dosing. Currently, LNPs and polymer-based systems are the most studied delivery platforms and will be discussed further. Other systems, including hybrid and inorganic nanoparticles, exosomes, cell-penetrating peptides, and protamines, are discussed elsewhere (Chabanovska et al., 2021; Klipp et al., 2023; Iqbal et al., 2024). 

LNPs represent the clinically most advanced platform for mRNA delivery. Ionizable LNPs were used to deliver viral mRNA in the approved vaccines against SARS-Cov2 (Polack et al., 2020; Baden et al., 2021). These are nanosized particles, composed primarily of lipids, arranged in a core-shell structure. Lipid components include (i) ionizable lipids, that remain neutral at physiological pH and become positively charged at low pH, facilitating mRNA encapsulation during formulation and promoting endosomal escape after cellular uptake; (ii) non-cationic (phospho)lipids that, besides stability, contribute to membrane fusion and increased cellular uptake; (iii) cholesterol, enhances nanoparticle stability and membrane integrity; (iv) polyethylene glycol (PEG)-conjugated lipids, which control nanoparticle size, prevent aggregation and prolong circulation time (Estapé Senti et al., 2024). The modular composition of LNPs allows extensive optimization to improve delivery efficiency and tolerability. However, important limitations remain, including preferential liver accumulation, limited extrahepatic targeting, and associated dose-dependent toxicity, which can restrict repeated administration.

Polymeric nanoparticles have emerged as promising alternatives to LNPs, particularly for extrahepatic delivery (Hamada et al., 2021; Yum et al., 2022), and have been associated with minimal proinflammatory responses in certain formulations (Uchida et al., 2013). These nanoparticles are biodegradable and biocompatible colloidal structures capable of encapsulating nucleic acids, including mRNA, for delivery into human cells. Cationic polymers such as PEI (polyethyleneimine) and PAMAM (polyamidoamine) dendrimers can electrostatically bind to negatively charged mRNA molecules, forming stable complexes, commonly referred to as polyplexes (Yang et al., 2023). Polymer nanoparticles offer additional advantages, including the ability to be lyophilized (freeze-dried) enabling long-term storage and stability, as well as high structural adaptability, which allows the delivery of diverse genetic cargoes. Their chemical versatility allows fine tuning of physicochemical properties customization and the development of targeted delivery systems. Nevertheless, polymer nanoparticle platforms have not yet reached scalable and reproducible manufacturing, mainly due to the challenge of maintaining the tightly controlled conditions used in laboratory-scale production when scaling up to pilot or industrial levels. This can cause batch-to-batch variability, affecting physicochemical properties of the nanoparticles, which can influence biodistribution, pharmacokinetics, cellular uptake, therapeutic efficacy, and safety (Zawidlak-Węgrzyńska and Rydz, 2026). In addition, lower transfection efficiency and potential cytotoxicity remain important limitations that require further optimization (Yang *et al.*, 2023;  Wang and Cheng, 2025).

Endosomal degradation is a major limiting step in mRNA delivery, as only a small fraction of internalized particles release mRNA into the cytosol. Ionizable LNPs generally exhibit higher endosomal escape efficiency because their lipids become positively charged in acidic endosomes, promoting membrane destabilization and cargo release. In contrast, polymeric carriers often rely on the “proton sponge” effect, which is consistently less efficient and may contribute to lower transfection rates. Although ionizable LNPs currently outperform most polymer systems in cytosolic delivery, ongoing polymer engineering aims to improve escape efficiency while reducing toxicity.

Moreover, the administration route is critical determinant for therapeutic success. For mRNA vaccine, intradermal and intramuscular injections are the most widely used routes, producing strong and durable immune responses, while relatively easy to administer (Pardi et al., 2015). Both approved COVID-19 mRNA-based vaccines were administered intramuscularly (Polack et al., 2020; Baden et al., 2021). Intravenous administration has also been explored but is less favored due to rapid liver accumulation and procedural complexity, except when hepatic protein expression is desired (Hosseini-Kharat et al., 2025). An administration route to avoid the blood-brain barrier is the intrathecal route, in which treatment is directly injected into the cerebrospinal fluid surrounding the spinal cord, offering a promising approach for neurological disorders (Monfrini et al., 2024). Less invasive alternatives are under investigation. The intranasal route is non-invasive, suitable for repeated administration and has the potential to access the central nervous system, via olfactory pathways (Dhaliwal et al., 2020). Additional approaches include the intratracheal aerosol-based method, oral mRNA delivery strategies, and intramyocardial injection. Ultimately, the best route for mRNA-drug administration depends on the therapeutic application, target tissue or organ, and the delivery platform employed. Continued comparative studies are essential to define the most effective and safest strategies for specific clinical applications.

## Advantages and limitations of mRNA-based therapies

The mRNA-based therapeutics have demonstrated remarkable potential for both disease prevention and clinical treatment (Wang et al., 2023). Their versatility has attracted significant interest from academic research groups and from the pharmaceutical industry, encompassing applications in immunotherapy as well as non-immunogenic strategies for the treatment of infectious diseases, cancer, and inherited genetic disorders (Weng et al., 2020). 

A defining feature of mRNA therapeutics is their exacerbated inflammatory properties, which acts as a double-edged sword. In cancer therapy, enhanced immune stimulation may be beneficial for the generation of robust antitumor responses. In contrast, for non-immunotherapeutic applications such as protein replacement therapies, increased immune activation is undesirable, as it may compromise treatment tolerability and limit sustained expression of therapeutic protein encoded by the mRNA (Shi et al., 2024). Consequently, the route of administration - including intravenous, subcutaneous, intradermal, intramuscular, intranasal, and intranodal- must be carefully selected, since it strongly influences biodistribution, antigen expression kinetics, and overall therapy efficacy (Zhang et al., 2019; Chaudhary et al., 2021). 

Unlike gene therapy protocols based on viral vectors or DNA molecules, mRNA does not enter the nucleus. Translation occurs directly in the cytoplasm, eliminating the risk of genomic integration, and thereby reducing the potential for insertional mutagenesis or long-term genetic instability, issues historically associated with some retroviral gene-delivery strategies (Cavazza et al., 2013; Parman et al., 2025). However, this safety advantage also introduces an important limitation: mRNA expression is inherently transient. As a result, repeated administrations are often required, particularly for protein-replacement therapies that demand sustained therapeutic protein levels.

When compared to DNA-based approaches, mRNA delivery offers several additional advantages. Once delivered into the cytoplasm, mRNA is directly translated by ribosomes, enabling rapid protein production. This feature shortens the onset of therapeutic activity and allows precise temporal control of protein expression (Tavernier et al., 2011; Qin et al., 2022). In contrast, plasmid DNA molecules must first enter the nucleus to undergo transcription before the mRNA can be exported back to the cytoplasm for translation. This additional intracellular step reduces efficiency, especially in non-dividing cells, where nuclear entry is limited (Youn and Chung, 2015). Consequently, DNA transfection often shows variable expression efficiency, whereas mRNA can be efficiently expressed across a wide range of cell types, including non-dividing cells (Andries et al., 2012).

*In vivo* delivery of mRNA-encoding monoclonal antibodies demonstrated detectable protein expression as early as two hours after administration, with peak expression occurring approximately two days later (Sabnis et al., 2018). By comparison, plasmid DNA expression may require up to two weeks to reach maximal levels and carries the theoretical risk of insertional mutagenesis (Deal et al., 2021). Thus, mRNA represents a faster and inherently safer platform for transient therapeutic protein production.

Figure 4 illustrates a direct comparison of DNA- and mRNA-mediated transfection using eGFP reporter protein expression. In this experiment, *in vitro* transcribed eGFP mRNA was compared with pcDNA.3.1-eGFP plasmid following liposome-mediated transfection (Lipofectamine 3000, Thermo Fisher Scientific) in human HEK293T cells. Fluorescence analysis (Figures 4A and 4B ) demonstrates a substantially higher proportion of eGFP expressing cells after mRNA transfection. Schematic representations of protein expression pathways following DNA or mRNA delivery are presented in Figures 4C and 4D .

**Figure 4 -:**
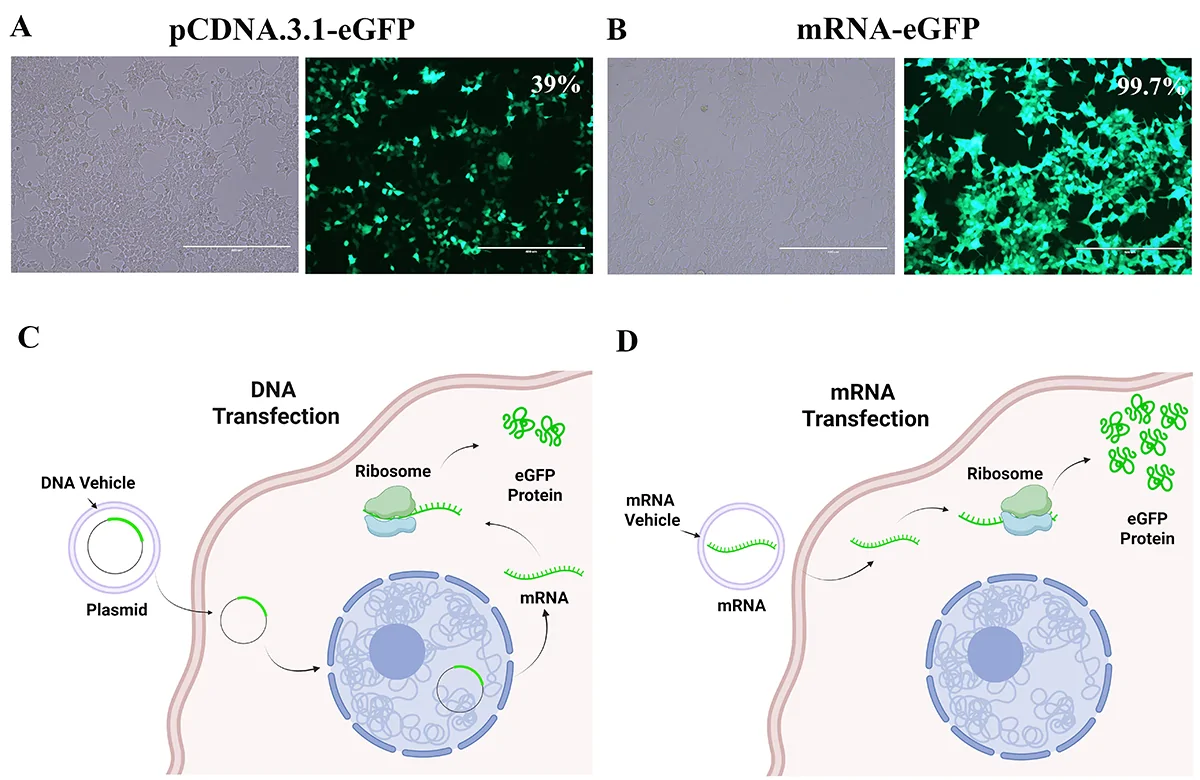
Comparison on the transfection into human cells of plasmid DNA or mRNA molecules. Analysis of eGFP protein expression after 24 hours of transfection with (A) plasmid DNA (pCDNA.3-eGFP) or (B) mRNA-eGFP by fluorescence microscopy. HEK293T cells were plated in 24-well and transfected with 250 ng of pCDNA.3.1-eGFP or *in vitro* produced mRNA encoding eGFP protein. The images were obtained with the same intensity and brightness settings by the Invitrogen EVOS microscope imaging system. The cell count and immunofluorescence intensity signal were obtained by the Image J2 software (Schindelin et al., 2012). Cells transfected with DNA showed a percentage of 39% of fluorescent cells, while 99.7% of cells transfected with mRNA showed efficient eGFP expression. Schematic representations of DNA (C) and mRNA (D) transfection through vehicle carriers. In (C)- liposome encapsulated DNA is delivered into the cytoplasm through vehicle-mediated fusion with the cell membrane, where it is released. The plasmid goes to the nucleus, where newly synthesized mRNA is processed and exported back to the cytoplasm. In the cytoplasm, the mRNA is translated into a protein by the ribosome. In (D)- liposome encapsulated mRNA is delivered into the cytoplasm through vehicle-mediated fusion with the cell membrane, where it is released directly in the cytoplasm and subsequently translated into a protein by the ribosome.

Overall, the expanding clinical success of mRNA-based technologies underscores the importance of understanding their biological mechanisms, delivery strategies and safety profiles. Continued advances in mRNA engineering, delivery platforms, and administration strategies are positioning mRNA therapeutics as a transformative modality in modern medicine, extending beyond vaccination to cancer immunotherapy, protein replacement therapies, and a broad spectrum of emerging biomedical applications.

## Potential applications of mRNA-based therapy for DNA repair genetic diseases

A major focus of our research interest is the application of mRNA-based technology to genetic disorders caused by defects on DNA repair pathways, particularly xeroderma pigmentosum (XP). These patients present deficiencies in nucleotide excision repair (NER) resulting from germinal mutations in genes encoding XPA-XPG or XPJ, which are required to remove bulky lesions from DNA, such as pyrimidine dimers induced by the ultraviolet (UV) component of sunlight. Alternatively, a variant form of XP patients (XPV) harbors mutations in POLH, encoding DNA polymerase eta, which is required for translesion synthesis and lesion bypass during replication. XP is a rare autosomal recessive disease characterized by extreme photosensitivity to sunlight, early onset of skin lesions, and a marked increased incidence of skin cancers in the sun-exposed areas, due to accumulation of unrepaired lesions and elevated somatic mutagenesis (Ain et al., 2021; Corradi et al., 2023; Yurchenko et al., 2023). Close to 20% of XP patients also develop progressive neurodegeneration and features of premature aging (Bradford et al., 2011; Menck and Munford, 2013). Other NER-associated disorders of interest include Cockayne syndrome (CS) and photosensitive Trichothiodystrophy (TTD). CS results from mutations in *CSA* and *CSB*, affecting transcription-coupled NER (TC-NER). Clinical manifestations include growth failure, neurodevelopmental impairment, microcephaly, sensorineural hearing loss, retinal degeneration, and photosensitivity without skin cancer predisposition (Laugel, 2013; Menck and Munford, 2013). TTD arises from mutations in *XPB*, *XPD* or *TTDA*, also impairing TC-NER. TTD is a multisystem condition characterized by developmental delay, ichthyosis, photosensitivity, recurrent infections, and presents sulfur-deficient brittle hair with a characteristic “tiger-tail” pattern under polarized microscopy (Lanzafame et al., 2025). 

Currently, no curative therapies are available for disorders, and clinical management relies largely on strict photoprotection and symptomatic treatment. mRNA-based technology emerges as a promising strategy, capable of transiently restoring expression of DNA repair proteins, alleviating disease manifestations. Two delivery strategies appear particularly promising. The first involves topical or intradermal administrations targeting the skin tissues. Because the skin is an accessible organ with high protein production capacity, localized mRNA delivery could restore DNA repair directly in affected keratinocytes (Ain et al., 2021). Although the stratum corneum represents a significant barrier to the transfer to macromolecule penetration, of large molecules, recent advances in the use of microneedle patches allow minimally invasive intradermal delivery of mRNA-based LNP formulations, enabling controlled and localized protein expression in skin cells (Zhang et al., 2026; Factor Bioscience). 

A second strategy aims to address neurological manifestations through central nervous system delivery via the olfactory pathway, therefore bypassing the blood-brain barrier (Dhaliwal et al., 2020). Proof-of-concept has been demonstrated with mucopolysaccharidosis I (MPS I), a lysosomal storage disease caused by deficiency in alpha-L-iduronidase (IDUA). Intranasal administration of liposome-encapsulated enzyme enabled delivery to the brain and peripheral organs (Schuh et al., 2024), while related studies achieved genome editing in the mouse brain using CRISPR/Cas9 plasmid delivery (Vera et al., 2022). Similar delivery platforms could potentially be adapted for mRNA encoding functional DNA repair proteins in XP, CS, or TTD patients. Collectively, these approaches highlight the potential of mRNA therapeutics as a versatile platform for treating DNA repair disorders. By enabling transient yet repeated restoration of defective proteins, mRNA-based therapies may improve survival, reduce disease burden, and significantly enhance the quality of life for individuals affected by these currently untreatable rare genetic conditions. Continued advances in delivery technologies and tissue targeting will be critical for translating this promising strategy into clinical reality.

## Future perspectives and remaining challenges in mRNA therapeutics

The rapid evolution of mRNA technology promises a revolution in modern medicine opening a route for innovative therapeutic molecules. The feasibility, scalability, and safety of this platform was demonstrated by the mRNA-based vaccines employed during the COVID19 pandemic. Other applications have expanded this technology toward further applications, including cancer immunotherapy and protein replacement therapies. Advances in RNA and delivery systems chemistry have the potential to increase the control of protein expression, tissue targeting, and immune modulation, with new avenues of research designed for translational medicine. 

These advances will provide solutions to the important challenges that still remain for mRNA-based therapies. Major areas of scientific investigation may provide the means for efficient delivery to different tissues, increase long-term safety particularly during repeated administration, improvement of storage stability, and control of immune activation. In addition, reducing manufacturing costs will be essential to ensure global accessibility to mRNA therapeutics, even for rare genetic and orphan diseases, which are historically neglected by pharmaceutical industries.

The rapid advances for mRNA-based therapies will nourish novel ideas and a cascade of uses further expanding the therapeutic landscape. As these technological and biological obstacles are progressively overcome, mRNA-based medicines may become a central pillar of precision medicine, offering adaptable, flexible, and highly specific therapeutic solutions for a wide spectrum of human diseases.

## Data Availability

 The data in this review come from published manuscripts, and the original mRNA transfection data are shown in Figure 4. Details can be provided upon request.
